# Dynamics of carbon and nitrogen storage in two typical plantation ecosystems of different stand ages on the Loess Plateau of China

**DOI:** 10.7717/peerj.7708

**Published:** 2019-09-18

**Authors:** Yanfang Wang, Ling Liu, Feixue Yue, Dong Li

**Affiliations:** 1 College of Agriculture, Henan University of Science and Technology, Luoyang, Henan, China; 2 State Key Laboratory of Soil Erosion and Dryland Farming on the Loess Plateau, Northwest Agriculture and Forestry University, Yangling, China

**Keywords:** Plantation forest, Ages, Soil organic carbon, Soil total nitrogen, Loess Plateau

## Abstract

In China’s Loess Plateau, afforestation and reforestation are considered the foremost practices for sequestering carbon and conserving soil and water. In order to evaluate the carbon storage changes of tree, soil, and litter, and the soil total nitrogen (STN) in two typical artificial forests in the region, we conducted plot surveys for different ages of both artificial forest types. Soil samples were collected at different depths from 0–100 cm. The results indicated that forest ecosystem carbon storage increased with tree development. The rates of mean annual carbon sequestration of *Pinus tabulaeformis* and *Robinia pseudoacacia* plantation ecosystems were 3.31 and 3.53 Mg ha^−1^ year^−1^, respectively. The rate of mean annual carbon sequestration of *R. pseudoacacia* plantation ecosystems was higher by 6.65% than that of *P. tabulaeformis* plantation ecosystems. The soil organic carbon (SOC) and STN decreased at deeper soil depths in both plantations at different stand ages, significantly decreasing in the 0–60 cm of soil (*P* < 0.05), and the highest SOC content and storage were in the top 0–20 cm of soil. The temporal patterns for SOC and STN changes at different soil sampling depths from 0 to 100 cm all showed an initial decrease during the early stage of restoration, and then an increase that coincided with the development of the two plantation forests. At 0–100 cm depth, the SOC storage was in the range of 40.95–106.79 and 45.13–113.61 Mg ha^−1^ for the *P. tabulaeformis* forest and *R. pseudoacacia* forest, respectively. The STN storage in the 0–100 cm soil layer with the stand age development ranged from 4.16 to 8.34 Mg ha^−1^ in the *R. pseudoacacia* plantation and 4.19–7.55 Mg ha^−1^ in the *P. tabulaeformis* forest. The results showed a significant positive correlation between SOC and STN. This study suggests that we should pay more attention to changes in soil carbon and nitrogen sequestration during long-term vegetation restoration.

## Introduction

It has been widely recognized that terrestrial ecosystems can release or absorb globally relevant greenhouse gases such as carbon dioxide (CO_2_), maintain the global carbon balance, and affect environment change (Heimann & Reichstein, 2008; Piao et al., 2018). Vegetation restoration on formerly degraded land is considered to be one of the most effective ways to improve carbon sinks in terrestrial ecosystems by improving the physical and chemical properties of degraded soil (Jia et al., 2005; Li, Niu & Luo, 2012). Plantations play an important role in improving the carbon sink of terrestrial ecosystems and have been widely recognized by the international community as an effective vegetation restoration measure (IPCC (Intergovernmental Panel on Climate Change), 2007). Thus, afforestation and reforestation have often been proposed as effective strategies for vegetation restoration and climate change mitigation (UNFCCC, 2005).

As the largest terrestrial ecosystem carbon stock, forest ecosystems store 50–60% of carbon in terrestrial ecosystems (Dixon et al., 1994; Post & Kwon, 2000). In particular, forest soil carbon sequestration plays a crucial role in the global carbon cycle, and accounts for 73% of the global soil carbon pool (Sombroek, Nachtergaele & Hebel, 1993). Carbon and nitrogen are important elements that maintain the structure, function, and stability of forest ecosystems. Soil nitrogen also plays a key role in carbon sinks by interacting with carbon for ecosystem productivity and carbon sequestration (Knops & Tilman, 2000; Reich et al., 2006). Studies have shown that dynamic change in soil nitrogen is the main factor influencing carbon sequestration in forest ecosystems (Luo et al., 2004). Therefore, assessing soil organic carbon (SOC) and soil total nitrogen (STN) storage dynamics in different forest types, stand ages and regions is crucial for the sustainable management of land resources and predictions of the future C and N cycles.

The Loess Plateau of China, an area of 643,105 km^2^, has very unique and deep loess and is considered one of the most severely eroded areas in the world (Peng, Wang & Yu, 1995). In the mid-20th century, people blindly pursuing economic interests and cut down a large number of forests, resulting in soil erosion and environmental damage (Deng, Liu & Shangguan, 2014). Revegetating degraded land is one of the principal strategies for soil erosion control and ecosystem recovery around the world (Deng, Wang & Shangguan, 2014). Since the 1960s, a series of national forestry projects have been carried out in China to counteract soil erosion and other environmental problems, including the Grain for Green program (GGP), which was implemented by converting low-yield sloped cropland, barren hills and wasteland into forest and grasslands across the country (Feng et al., 2013; Jia et al., 2014; Wang, Liu & Shangguan, 2017). The Loess Plateau pioneered the implementation of the GGP in China. Afforestation and reforestation conservation projects have gradually increased carbon storage in forest ecosystems (Wang, Liu & Shangguan, 2018).

*Robinia pseudoacacia* is the main afforestation tree species in the loess hilly region and has the largest artificial forest area due to its strong roots, rapid growth, drought tolerance, and high survival rate. *Pinus tabulaeformis* is the warm temperate coniferous tree species in the sub-humid and semiarid regions of the Loess Plateau and has beneficial ecological functions such as soil and water conservation. A series of afforestation projects in the Loess Plateau have been carried out, such as the implementation of the GGP in which large-scale pure artificial *P. tabulaeformis* and *R. pseudoacacia* forests were planted in the region. These two tree species were planted in large areas of different stand ages at Shanzhang Forest Farm on the Loess Plateau. The afforestation area of *R. pseudoacacia* and *P. tabulaeformis* accounted for 32% and 28%, respectively, of the total afforestation area of Shanzhang Forest Farm on the Loess Plateau. Until now, most studies have focused on plant biomass, soil carbon and nitrogen following vegetation restoration on the loessal soil of the north–central Loess Plateau (Wang, Liu & Xu, 2009; Deng, Shangguan & Sweeney, 2013; Zhang et al., 2013; Deng, Wang & Shangguan, 2014). The Shanzhang Forest Farm is located on the southeast margin of the Loess Plateau, with a cinnamon soil type (Eutric Luvisd, FAO soil taxonomy) (Gao, 2013).

Several studies have assessed SOC and STN dynamics in response to land use changes on the Loess Plateau (Jia et al., 2012; Zhang et al., 2013; Deng, Liu & Shangguan, 2014; Liu et al., 2014). Additionally, some investigations have studied carbon and nitrogen accumulation in typical plantations at different stand ages and at various soil layers (Wang, Liu & Xu, 2009; Singh et al., 2012; Gao et al., 2014; Chen et al., 2017). However, little is known about long-term changes to SOC and STN in *P. tabulaeformis* and *R. pseudoacacia* plantations that were planted on cinnamon soil in the deeper soil layers on the Loess Plateau.

Therefore, the objectives of this study were (1) to evaluate the distribution of the carbon storages of plant, litter, and soil in *P. tabulaeformis* and *R. pseudoacacia* plantation ecosystems; and (2) to investigate the dynamic changes of SOC and STN in the 0–100 cm soil layer under different stand ages in two artificial forest ecosystems on the Loess Plateau of China.

## Materials and Methods

### Study area

**Figure 1:**
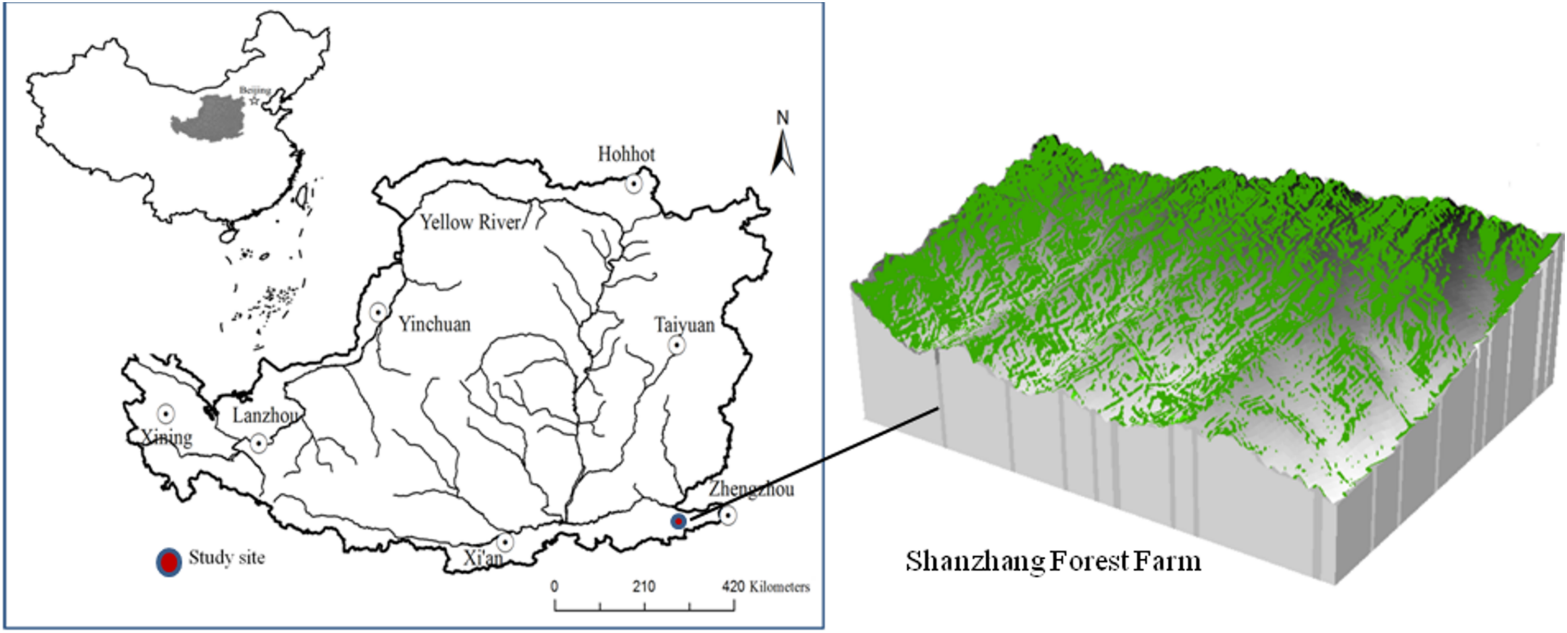
Location of the Shanzhang Forest Farm in the Loess Plateau.

The study was conducted in the Shanzhang Forest Farm of Yanshi City, Henan Province (34°29′27″–34°33′09″N, 112°43′15″–112°46′45″ E), located on the southeast margin of the Loess Plateau, China, covering a total area of 1,466.67 ha (Fig. 1). The area has a warm temperate continental monsoon climate. The soil type is cinnamon soil (Eutric Luvisd, FAO soil taxonomy) with a pH of 7.82–8.31 (Gao, 2013). The altitude of the region averages 700 m, the mean annual temperature is 14.2 °C, the mean annual precipitation is 500–600 mm, and the annual frost-free period is 211 days. The actual sunshine hours for the year were 2,248.3 h, and the percentage of sunshine days for the year was 51%.

According to historical records and information provided by forest farm staff, *P. tabulaeformis* and *R. pseudoacacia* forests have been planted in this area since the 1960s for soil erosion control and ecosystem recovery. These two types of forests are typical artificial forest ecosystems with various ages. The *R. pseudoacacia* plantations in this region were 8, 20, 38, and 58 years old, while the *P. tabulaeformis* plantations were 6, 15, 35, and 56 years old. There are sparse shrubs and herbaceous plants with little biomass beneath the trees. The cinnamon soil (Eutric Luvisd, FAO soil taxonomy) was developed on carbonate parent material and has obvious adhesion and calcification. Basic information about soil properties for the surface soil (0–20 cm) of the different tree types and stands is shown in Table 1.

**Table 1:** pH, soil bulk density, and soil particle distribution in the 0–20 cm soil layer in different trees.

Tree species	Stand age (year)	pH (mean ± SD)	Bulk density (g cm^−3^) (mean ± SD)	Clay (%)	Silt (%)	Sand (%)
*Pinus tabulaeformis*	6	7.73 ± 0.15	1.37 ± 0.12	25.76	38.59	35.65
	15	7.56 ± 0.09	1.30 ± 0.14	29.64	38.89	31.47
	35	7.27 ± 0.13	1.25 ± 0.09	24.53	37.30	38.17
	56	7.13 ± 0.16	1.18 ± 0.07	24.90	37.90	37.20
*Robinia pseudoacacia*	8	7.85 ± 0.12	1.32 ± 0.08	24.15	32.44	43.41
	20	7.88 ± 0.08	1.28 ± 0.11	20.33	32.90	46.78
	38	7.71 ± 0.10	1.25 ± 0.09	23.10	35.67	41.24
	58	7.37 ± 0.11	1.19 ± 0.06	23.98	33.65	42.37
Farmland	–	7.93 ± 0.09	1.23 ± 0.08	18.81	32.24	48.95
	–	7.89 ± 0.12	1.20 ± 0.07	20.34	30.87	48.79
	–	7.91 ± 0.08	1.19 ± 0.11	25.59	38.41	36.00

### Field sampling and measurements

Field surveys took place between July 5 and August 10, 2017. According to the distribution of forest areas in different afforestation periods provided by forest farm workers, we selected representative plots that were far from the forest edge. The plots were no more than two km apart, and the plot characteristics, such as slope, aspect, altitude, and soil type, were very similar (Table 2). Since we focused on the conversion from sloped farmland to plantation, we selected three nearby maize (*Zea mays*) plots as 0-year-old stands for comparison. The farmland was planted as a wheat-maize rotation. The average amount of fertilizer applied was 200–250 kg ha^−1^ of sheep manure as the base fertilizer in October of the winter wheat season, and 200–300 kg ha^−1^ of urea applied in April as topdressing. In addition, 100–200 kg ha^−1^ of urea was applied in August as topdressing in the maize season.

**Table 2:** Information about sample plots.

Tree species	Stand age (year)	Latitude (N)	Longitude (E)	Altitude (m)	Aspect (°)	Slope (°)	Stand density (plants ha^−1^)	DBH (cm) (mean ± SD)	Height (m) (mean ± SD)
*Pinus* *tabulaeformis*	6	34°30′25.2″	112°42′46.8″	575.3	NW55	18	1,970	2.31 ± 0.41	2.52 ± 0.44
	15	34°30′43.2″	112°43′40.8″	589.5	NW67	20	1,865	6.39 ± 2.11	6.12 ± 2.32
	35	34°30′21.6″	112°43′55.2″	595.7	NW65	25	1,745	13.31 ± 3.51	12.19 ± 2.76
	56	34°30′36.0″	112°43′12.0″	595.3	NW65	25	1,355	19.08 ± 3.13	16.09 ± 3.54
*Robinia pseudoacacia*	8	34°30′21.6″	112°43′4.8″	509.8	NE45	28	1,770	3.92 ± 0.35	5.52 ± 1.01
	26	34°30′46.8″	112°43′22.8″	513.5	NE65	37	1,650	9.23 ± 2.72	9.91 ± 2.11
	38	34°30′28.8″	112°43′44.4″	515.8	NE59	35	1,550	18.25 ± 3.18	15.10 ± 3.23
	58	34°30′43.2″	112°43′30.0″	566.9	NW60	32	1,435	25.20 ± 4.39	19.10 ± 3.76
Farmland	0	34°31′1.2″	112°43′8.4″	339.7	NE40	12			
	0	34°31′8.4″	112°43′19.2″	345.6	NW35	15			
	0	34°31′43.2″	112°43′19.2″	338.9	NW42	10			

Five plots of 20 × 20 m in the forest communities were chosen for different tree species in each stand age. The number, height, and stem diameter at breast height (DBH) for all trees with DBH ≥ 5 cm (DBH ≥ 2 cm for young-aged trees) were recorded within each plot. The tree density, average tree height, and DBH at different ages were calculated. The information about the plots is presented in Table 2. To avoid damaging the biomass caused by felling trees, we used allometric growth equations specifically for *P. tabulaeformis* and *R. pseudoacacia* forests (built from studies in regions with similar soil properties and environmental conditions) to determine the biomass of the plantation forests (Table 3) (China’s State Forestry Administration, 2011). Since shrubs and herbs are sparse beneath the trees and have little biomass, we did not measure them. Five 1 × 1 m litter samples were selected along a diagonal line in each forest community. All litter in the sample was collected and weighed as fresh weight, taken to the laboratory and put in a 70 °C oven to dry to a constant weight. After weighing, the dry weight of litter in the sample was obtained, and the litter amount per unit area was obtained.

**Table 3:** The biomass allometric equations with variances of diameter at breast height (DBH) and height (H) of *Pinus tabulaeformis* and *Robinia pseudoacacia*.

Tree species	Organ	Biomass allometric equation	*R*^2^
*Pinus tabulaeformis*	Trunk	W_T_ = 0.027636(D^2^H)^0.9905^	0.9908
	Branch	W_B_ = 0.0091313(D^2^H)^0.982^	0.9171
	Foliage	W_F_ = 0.0045755(D^2^H)^0.9894^	0.9984
	Root	W_R_ = 0.0084800(D^2^H)^0.988^	0.9930
*Robinia pseudoacacia*	Trunk	W_T_ = 0.02583(D^2^H)^0.95405^	0.9899
	Branch	W_B_ = 0.00464(D^2^H)^3.21307^	0.9782
	Foliage	W_F_ = 0.02340(D^2^H)^1.92768^	0.9546
	Root	W_R_ = 0.01779(D^2^H)^2.64480^	0.9397

**Note:**

The biomass allometric equations were adopted from “Carbon sink metering and monitoring guidelines of afforestation projects,” published by China’s State Forestry Administration (2011).

Soil samples were taken at the four corners and the centre of each plot using a soil drilling sampler (five cm diameter). In each sample plot, the litter on the ground was removed before sampling, and soil samples representing depths of 0–20, 20–40, 40–60, 60–80, and 80–100 cm were taken at the five points and mixed to make a representative sample for each soil layer. All soil samples were sieved through a two mm screen, and roots and other debris were removed. Each sample was air-dried and stored at room temperature for the determination of SOC and STN contents. The soil bulk density (BD) (g cm^−3^) of the different soil layers (0–20, 20–40, 40–60, 60–80, and 80–100 cm) was measured using a soil bulk sampler with a 5.0 cm diameter and 5.0 cm high stainless steel cutting ring (three replicates) at points adjacent to the soil sampling plots. The original volume of each soil core and their dry mass weight after oven–drying at 105 °C for 48 h were used to calculate the soil BD.

### Physical and chemical analysis

Soil BD was calculated based on the inner diameter of the core sampler, the sampling depth and the oven–dried weight of the composite soil samples (Jia et al., 2005). Soil pH was measured in distilled water mixed 5:1 (by mass) with dry soil using a Delta 320 pH meter (Mettler–Toledo Instruments (Shanghai) Ltd, Shanghai, China) equipped with a calibrated combined glass electrode. SOC and litter carbon content were assayed by dichromate oxidation (Kalembasa & Jenkinson, 1973), and STN concentration was assayed using the Kjeldahl method (Jackson, 1973). A laser particle analyzer operating over a range of 0.02–2,000 μm (Mastersizer 2000 particle size analyzer, Malvern Instruments, Ltd., Malvern, UK), based on the laser diffraction technique, was used to measure particle size.

### Calculation of carbon storage in tree and litter layers

The carbon storage of tree vegetation and litter layers can be calculated by multiplying the biomass values by their relevant carbon fractions (*C_f_*). In this study, we used the biomass growth equation for the two plantations (Table 3) to estimate the living tree biomass. The carbon fractions (*C_f_*) of *R. pseudoacacia* and *P. tabulaeformis* are 0.48 and 0.52, respectively (Li & Lei, 2010). The carbon storage of the tree and litter layers was calculated using the following equation:

(1) ${C_v}{\rm{\,=\,}}B \times {C_f}$

where *C_v_* is the carbon storage in tree and litter layers (Mg ha^−1^); *B* is the biomass of the tree and litter (Mg ha^−1^); and *C_f_* is the carbon fraction.

### Calculation of soil carbon and nitrogen storage

Soil organic carbon storage was calculated according to the SOC content of the soil layer, its soil BD and sampling depth (Li et al., 2015). Coarse fractions (>2 mm) were very rare in the soil samples. Therefore, the study used the following formula to calculate SOC storage (*Cs*) (Guo & Gifford, 2002; Deng, Shangguan & Sweeney, 2013):

(2) ${C_{{\rm{si}}}}{\rm{\,=\,B}}{{\rm{D}}_i} \times {\rm{SO}}{{\rm{C}}_i} \times {D_i}{\rm{/10}}$

where *C*_si_ is SOC storage (Mg ha^−1^) in the soil layer *i*; BD*_i_* is the soil BD in soil layer *i* (g cm^−3^); SOC*_i_* is the SOC concentration of the *i*th soil layer (g kg^−1^); *D_i_* is the soil thickness of the *i*th soil layer (cm).

Equation (3) was used to calculate STN storage (Rytter, 2012; Deng, Shangguan & Sweeney, 2013):

(3) $${N_{{\rm{si}}}}{\rm{\,=\,}}\,{\rm{B}}{{\rm{D}}_i} \times {\rm{ST}}{{\rm{N}}_i} \times {{{D}}_i}{\rm{/10}}$$

where *N*_si_ is STN storage (Mg ha^−1^) in the soil layer *i*; BD*_i_* is the soil BD of the soil layer *i* (g cm^−3^); STN*_i_* is the STN concentration of the soil layer *i* (g kg^−1^); *D_i_* is the soil thickness of the soil layer *i* (cm).

In this study, the carbon storage of the artificial forest ecosystems is the sum of tree biomass carbon, litter carbon and SOC.

### Statistical analysis

One-way ANOVA was used to analyze the differences in SOC content, SOC storage, STN content, and STN storage in the same soil layers among the different stand ages. Differences were evaluated at *P* = 0.05. A generalized linear model was used to carry out the multiple comparisons. Pearson’s test was used to determine whether there were significant correlations between SOC and STN. All statistical analyses were performed using the software program SPSS, ver. 17.0 (SPSS Inc., Chicago, IL, USA), and all processed data were converted into images using the Origin software program, ver. 8.0.

## Results

### Distribution pattern of carbon storage in *P. tabulaeformis* and *R. pseudoacacia* plantation ecosystems

The carbon storage of the *P. tabulaeformis* plantation ecosystem increased from 46.46 Mg ha^−1^ in the 6-year-old to 211.84 Mg ha^−1^ in the 56-year-old (Table 4). The carbon storage in tree biomass and litter of *P. tabulaeformis* significantly increased from 0.67 to 86.88 Mg ha^−1^ and 0.52 to 4.99 Mg ha^−1^, respectively. The storage of SOC increased from 45.27 Mg ha^−1^ in the 6-year-old to 119.97 Mg ha^−1^ in the 56-year-old (Table 4).

**Table 4:** Allocation of the carbon pool of different forest types.

Tree species	Stand age (year)	Carbon storage in trees (Mg ha^−1^)	Carbon storage in litter (Mg ha^−1^)	Carbon storage in soil (Mg ha^−1^)	Carbon storage in ecosystem (Mg ha^−1^)	Fraction of soil carbon (%)
*Pinus tabulaeformis*	6	0.67	0.52	45.27	46.46	97.44
	15	11.32	2.29	55.57	69.18	80.33
	35	56.18	3.30	85.99	145.47	59.11
	56	86.88	4.99	119.97	211.84	56.63
*Robinia pseudoacacia*	8	2.17	0.73	46.88	49.78	94.17
	20	17.26	2.24	63.29	82.79	76.45
	38	61.89	3.95	88.45	154.29	57.33
	58	92.91	5.82	127.61	226.34	56.38
Farmland	–	–	–	49.22	49.22	100
	–	–	–	50.23	50.23	100
	–	–	–	48.39	48.39	100

In the *R. pseudoacacia* plantation ecosystem, carbon storage increased from 49.78 Mg ha^−1^ in the 8-year-old stand to 226.34 Mg ha^−1^ in the 58-year-old stand (Table 4). The carbon storage in trees, litter, and soil increased from 2.17 to 92.91 Mg ha^−1^, 0.73 to 5.82 Mg ha^−1^, and 46.88 to 127.61 Mg ha^−1^, respectively. SOC in the *R. pseudoacacia* plantation ecosystem accounted for 56.38–94.17% of forest ecosystem carbon storage (Table 4). The carbon storage in trees and soil played a major role in these two typical plantation ecosystem carbon sinks, notably through their soil carbon sequestration.

The mean annual carbon sequestration rates of *P. tabulaeformis* and *R. pseudoacacia* plantation ecosystems were 3.31 and 3.53 Mg ha^−1^ year^−1^, respectively. The mean annual carbon sequestration rate of the *R. pseudoacacia* plantation ecosystems was 6.65% higher than that of the *P. tabulaeformis* plantation ecosystems. There was no significant difference between the two plantations.

### Content and storage of SOC in the two plantations

**Figure 2:**
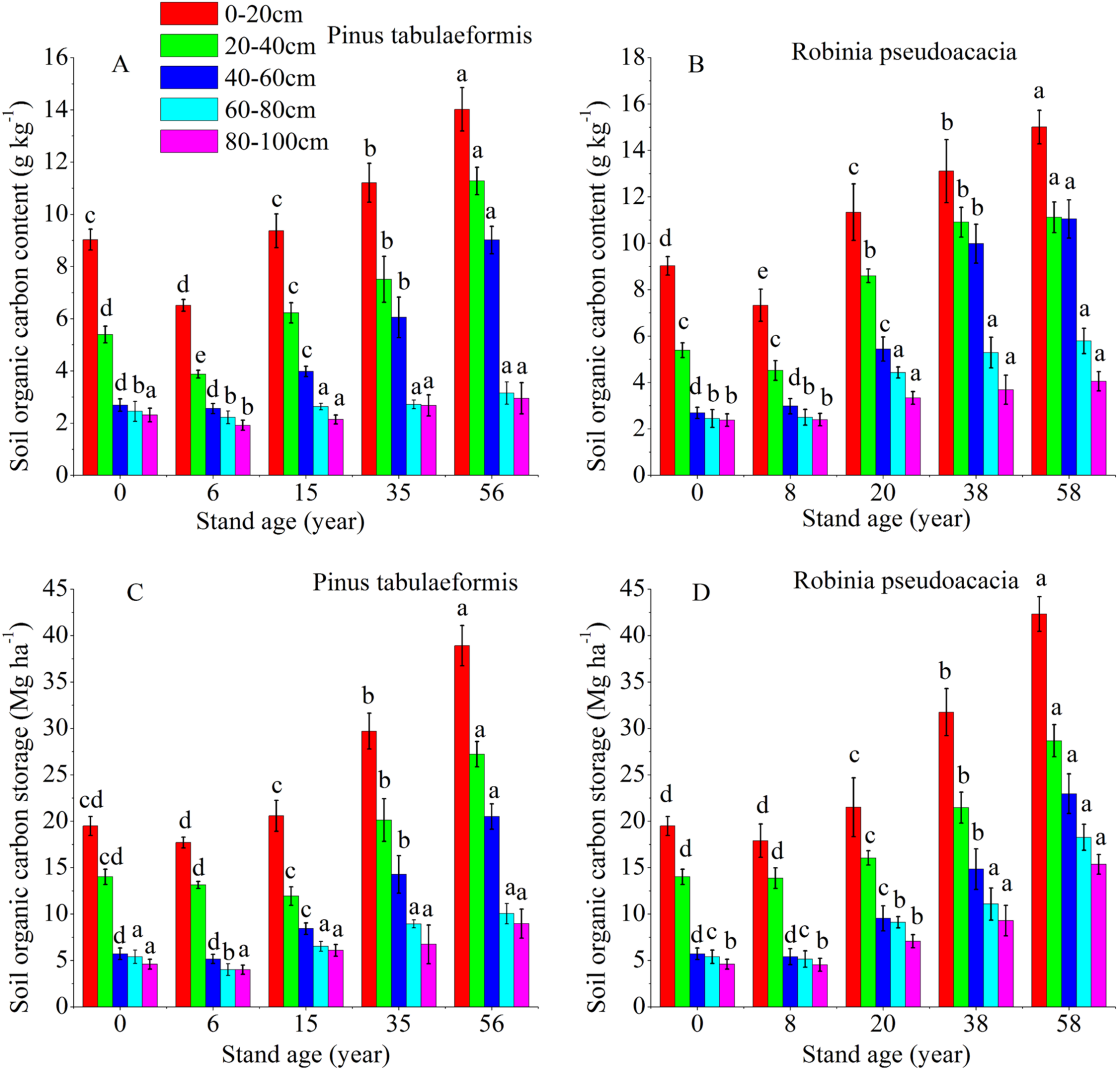
Soil organic carbon content and organic carbon storage in the different soil layers at each stand age of *Pinus tabulaeformis* and *Robinia pseudoacacia*. (A) Soil organic carbon content in *Pinus tabulaeformis*. (B) Soil organic carbon content in *Robinia pseudoacacia*. (C) Soil organic carbon storage in *Pinus tabulaeformis*. (D) Soil organic carbon storage in *Robinia pseudoacacia*. Note: Different letters indicate significant differences in the same soil layer of different stand ages at the 0.05 level

The temporal pattern for SOC content and storage in the 0–20, 20–40, 40–60, 60–80, and 80–100 cm soil layers all showed an initial decrease during the early stage (<6 and 8 years), and then an increase in carbon coinciding with the development of the two plantation forests (Fig. 2). In both forests, the SOC storage and content drastically decreased in the 0–60 cm profile. Below 60 cm, there was no significant difference in carbon content and storage for both plantations’ development (Fig. 2). The SOC content and storage decreased gradually at deeper soil layers in the same forest age. SOC mainly accumulated in the topsoil layer (0–20 and 20–40 cm); notably, the highest SOC content and storage were in the 0–20 cm topsoil. The SOC storage was in the range of 15.61–39.97 Mg ha^−1^ at depths of 0–20 cm and 40.95–106.79 Mg ha^−1^ at 0–100 cm for the *P. tabulaeformis* forest, while the SOC storage was in the range of 16.91–42.33 Mg ha^−1^ at depths of 0–20 cm and 45.13–113.61 Mg ha^−1^ at 0–100 cm for the *R. pseudoacacia* forest.

The SOC content in the *R. pseudoacacia* plantation was higher than that of the *P. tabulaeformis* plantation in the five soil layers at similar ages. For the *R. pseudoacacia* plantation, the SOC content at 0–100 cm averaged 3.94, 6.63, 8.58, and 9.41 g kg^−1^ for the 8-, 20-, 38- and 58-year-old, respectively. For the *P. tabulaeformis* plantation, the SOC content at 0–100 cm averaged 3.42, 4.87, 6.03, and 8.08 g kg^−1^ for the 6-, 15-, 35- and 56-year-old, respectively.

### Content and storage of STN in the two types of plantations

**Figure 3:**
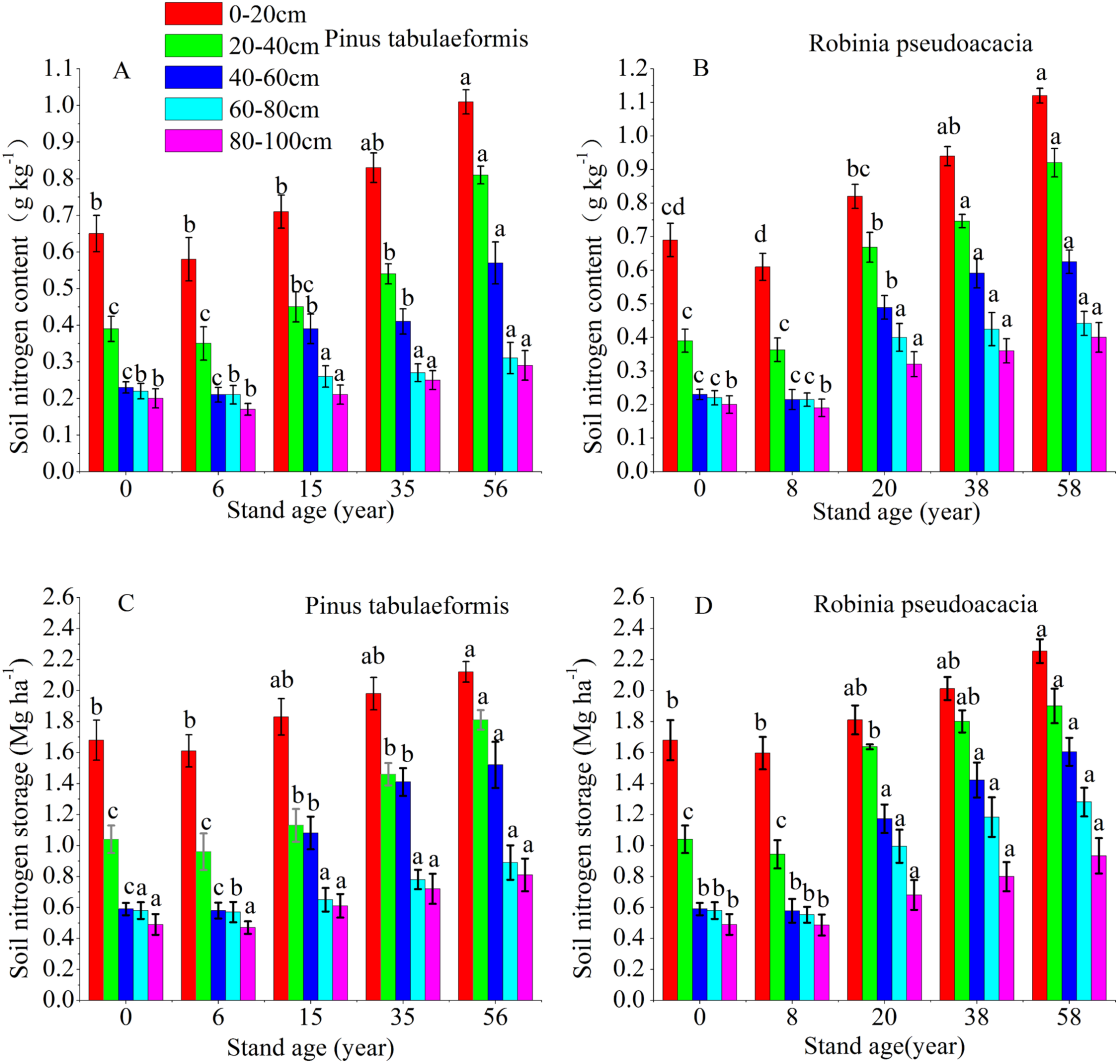
Soil nitrogen content and storage in the different soil layers at each stand age of *Pinus tabulaeformis* and *Robinia pseudoacacia*. (A) Soil nitrogen content in *Pinus tabulaeformis*. (B) Soil nitrogen content in *Robinia pseudoacacia*. (C) Soil nitrogen storage in *Pinus tabulaeformis*. (D) Soil nitrogen storage in *Robinia pseudoacacia*. Note: Different letters indicate significant differences in the same soil layer of different stand ages at the 0.05 level.**

The vertical pattern of STN content and storage closely followed the distribution of SOC, that is, the 0–60 cm soil layer decreased sharply, and the deep soil (60–100 cm) tended to be stable (Fig. 3). The soil nitrogen content and storage of the same forest age decreased at deeper soil layers. The STN content and storage at 0–20, 20–40, 40–60, 60–80, and 80–100 cm soil layers of the 6-year-old *P. tabulaeformis* and the 8-year-old *R. pseudoacacia* were all lower than that of farmland. The STN content and storage at the same soil layer decreased in the initial stage after tree planting and then increased with the development of the two plantations (Fig. 3).

The STN content and storage in the *R. pseudoacacia* forest were higher than that in the *P. tabulaeformis* plantation at the approximate forest age and same soil layer. However, the differences in STN content and storage were not significant between the two forests in the deep soil layers (60–80, 80–100 cm). In the *P. tabulaeformis* plantation, the average STN content in the 0–100 cm soil layer at different stand ages was 0.30, 0.40, 0.48, and 0.62 g kg^−1^ for the 6-, 15-, 35- and 56-year-old stands, respectively. In the *R. pseudoacacia* plantation, it was 0.32, 0.54, 0.67, and 0.74 g kg^−1^ for the 8-, 20-, 38- and 58-year-old stands, respectively. The average STN storage in the 0–100 cm soil layer at different stand ages was 4.16, 6.49, 7.63, and 8.34 Mg ha^−1^ for the 8-, 20-, 38- and 58-year-olds, respectively, in the *R. pseudoacacia* plantation and 4.19, 5.30, 6.35, and 7.55 Mg ha^−1^ for the 6-, 15-, 35- and 56-year-olds, respectively, in the *P. tabulaeformis* forest.

### C/N of the two types of plantations

**Figure 4:**
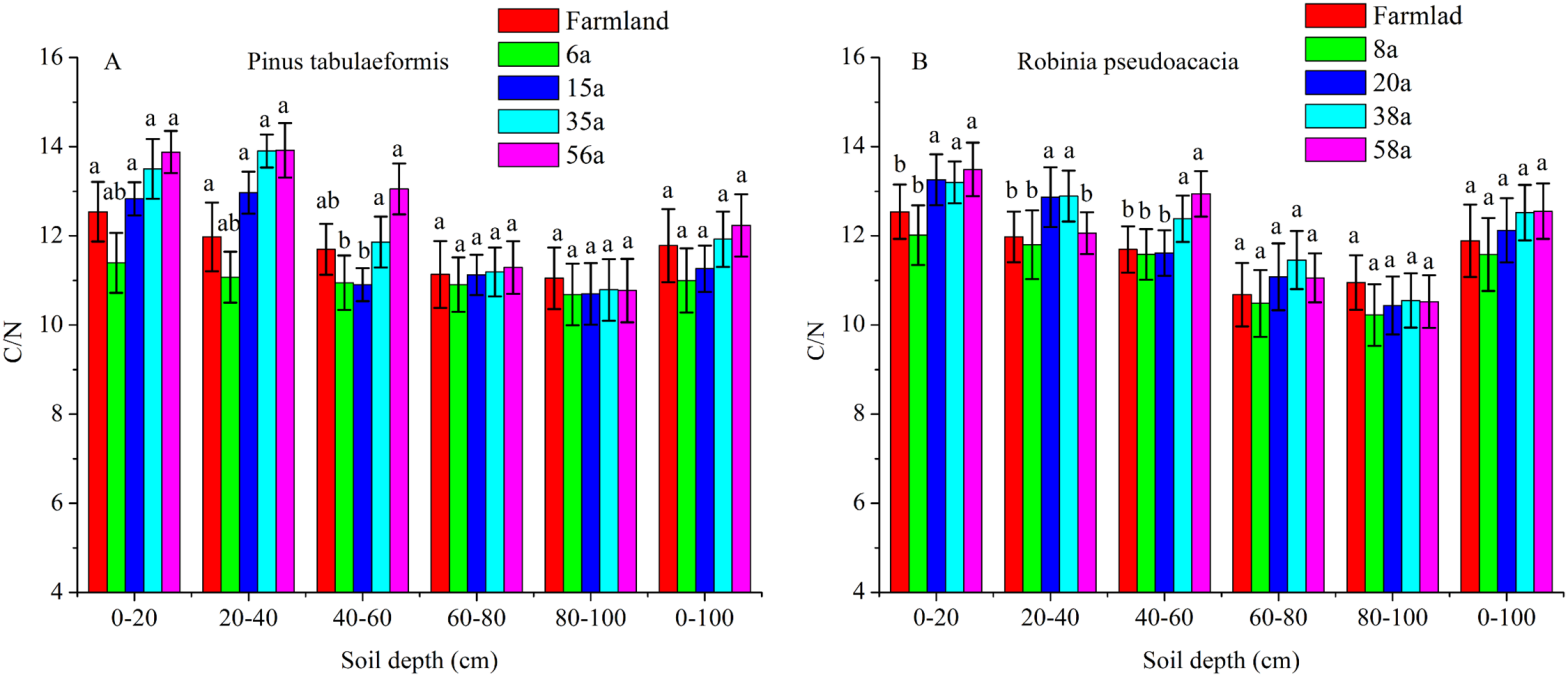
The C/N ratios in different soil layers at each stand age of *Pinus tabulaeformis* and *Robinia pseudoacacia*. (A) The C/N ratios in *Pinus tabulaeformis*. (B) The C/N ratios in *Robinia pseudoacacia*. Note: Different letters indicate significant differences in the same stand ages of different soil layers at the 0.05 level.

The C/N ratios calculated as SOC/STN under various soil layers and forest ages in the *P. tabulaeformis* plantation ranged from 10.06 to 13.88, and from 10.12 to 13.49 for the *R. pseudoacacia* plantation. Generally, the C/N ratios were higher in shallow soil (0–20, 20–40, 40–60 cm) than in deeper soil (60–80, 80–100 cm) in the same stand ages. The C/N ratios were not significantly different with the restoration of both forests at the 60–80 and 80–100 cm soil layers (*P* > 0.05) (Fig. 4).

The C/N ratios in the 0–20, 20–40, and 40–60 cm soil layers were higher in the *R. pseudoacacia* plantation than in the *P. tabulaeformis* plantation. This result was consistent with the much higher SOC contents in the *R. pseudoacacia* plantation. In general, the C/N ratios at 0–100 cm did not vary significantly at different stand ages (Fig. 4).

**Figure 5:**
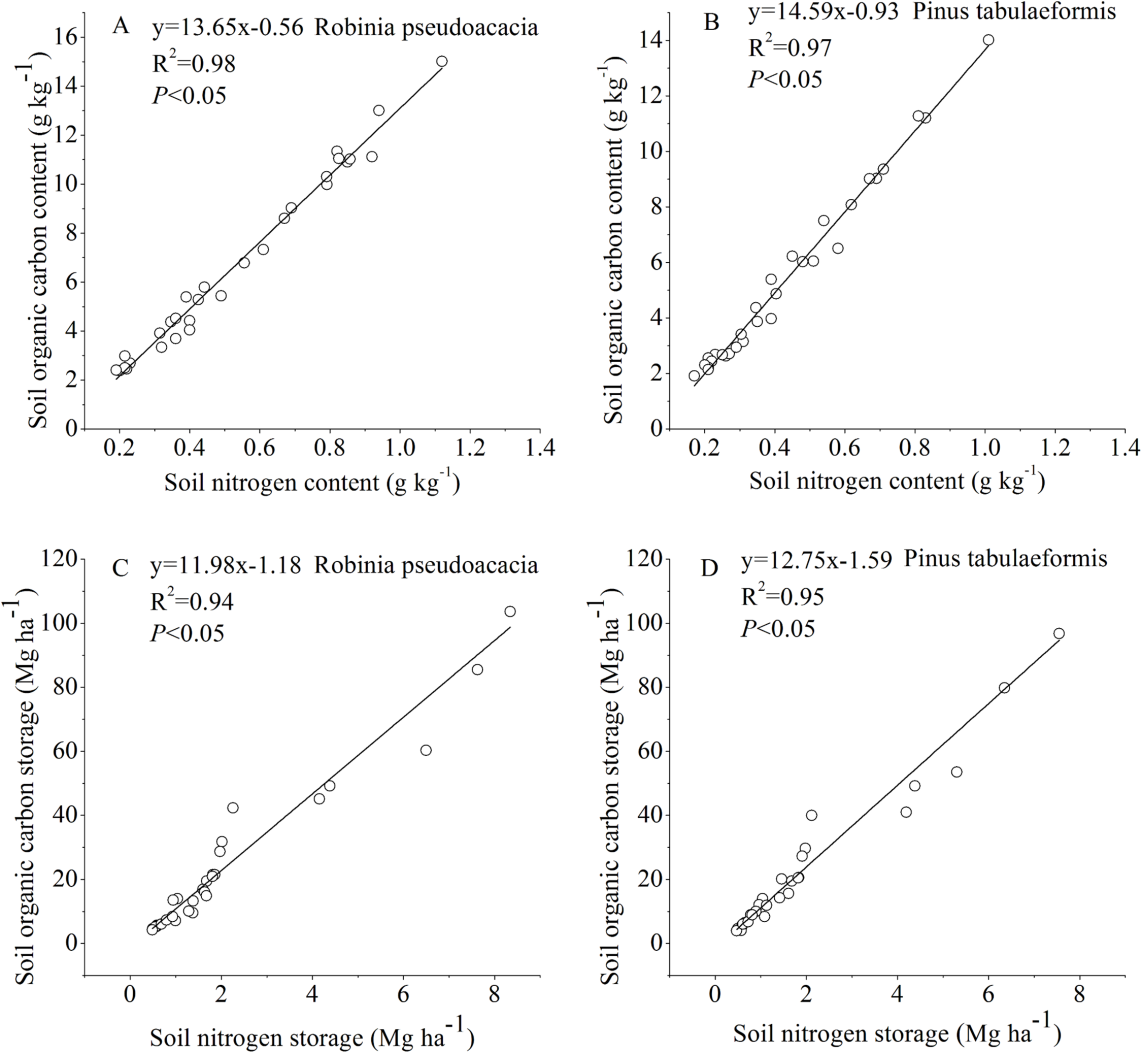
Relationship between soil organic carbon and soil nitrogen. (A) Relationship between soil organic carbon content and soil nitrogen content in *Robinia pseudoacacia*. (B) Relationship between soil organic carbon content and soil nitrogen content in *Pinus tabulaeformis*. (C) Relationship between soil organic carbon storage and soil nitrogen storage in *Robinia pseudoacacia*. (D) Relationship between soil organic carbon storage and soil nitrogen storage in *Pinus tabulaeformis*.

The SOC and STN for the *R. pseudoacacia* and *P. tabulaeformis* forests at different stand ages were linearly fitted (Fig. 5). The results showed that there was a significant positive correlation between SOC and STN (*P* < 0.05). The soil chemical substances and physical properties changed during the tree development process.

## Discussion

### The vegetation carbon storage in the two plantations

This study showed that the carbon storage of tree and litter biomass increased significantly with the development of both plantations. There are several reasons for this increase of carbon storage in tree and litter biomass. First, forest age is an important factor determining forest vegetation carbon sequestration (Guo & Gifford, 2002; Yang, Luo & Finzi, 2011; Li, Niu & Luo, 2012). The accumulation of vegetation biomass carbon along the age gradient could be ascribed to the increase in leaf area index before canopy closure (Sprugel, 1984). In this study, these plantations grew into mature forests with high carbon densities, causing the carbon storage of forest vegetation to increase. Second, planted forests are usually well managed and preserved with good grow, more vegetation and litter biomass have strong carbon sequestration capacity. In eastern Canada, biomass carbon increased with vegetation restoration time for up to 50 years, and the carbon sequestration ability of planted trees was higher than that of natural vegetation restoration (Tremblay & Ouimet, 2013). Wang, Liu & Shangguan (2017) also reported that plantations had higher carbon sequestration than natural restoration within a time scale of decades on the Loess Plateau.

The mean carbon sequestration rates of the tree layer in the *P. tabulaeformis* and *R. pseudoacacia* plantations were 1.72 and 1.81 Mg ha^−1^ year^−1^, respectively. These were 20.28% and 26.57% higher than that of the vegetation of forests in the warm temperate zone, which was 1.43 Mg ha^−1^ year^−1^ (Wu et al., 2008). The carbon storage in the trees and litter of the *R. pseudoacacia* plantation was higher than that of the *P. tabulaeformis* plantation, which may be due to the difference of tree growth characteristics and the environment.

### SOC and STN in the two forests

When compared with the carbon sequestration in vegetation biomass, the mechanism of soil carbon sequestration with vegetation restoration is more complex. As far as current studies have been concerned, there are four main temporal patterns of soil carbon sequestration resulting after converting degraded farmlands to forests: (1) With the growth of trees, the soil carbon storage is almost unchanged (Sartori et al., 2007); (2) the soil carbon storage decreases with increasing forest age (Kirschbaum, Guo & Gifford, 2008); (3) the soil carbon storage increases with increasing tree age (Deng et al., 2013; Dou et al., 2013); and (4) soil carbon initially decreases during the early stage, and then gradually increases to net C gains (Zhang et al., 2010; Lu et al., 2013; Deng, Wang & Shangguan, 2014; Chen et al., 2017). Our results are basically consistent with those of the fourth pattern of soil carbon sequestration. The temporal patterns for soil carbon change in different soil layers of the *P. tabulaeformis* and *R. pseudoacacia* plantations all showed an initial decrease in the early stage, and then increased to obtain net carbon coinciding with forest development. Lu et al. (2013) also reported that the SOC of *R. pseudoacacia* forests planted on the Loess Plateau of China would change from a decrease to an increase in 10–15 years.

The change of SOC during forest development is determined by the balance between carbon input and output. In the early stage of afforestation, the soil carbon output was far greater than the soil carbon input. This is because of the disturbance of soil, the lower productivity of new vegetation, the scarcity of leaf litter, and a low root biomass. This resulted in the depletion of SOC in the early stage of afforestation (Don et al., 2009; Li, Niu & Luo, 2012). Litter fall in *P. tabulaeformis* and *R. pseudoacacia* plantations increased with the development of forest stand, and the annual soil respiration was much lower (Chen et al., 2017; Yang et al., 2007). This resulted in soil carbon accumulation at the older forest stage with the development of forest stand.

The temporal pattern for STN storages exhibited similar trends to SOC following the forest conversion. Our results were consistent with past studies (Noh et al., 2010; Li, Niu & Luo, 2012; Chen et al., 2017). Li, Niu & Luo (2012) found that soil carbon and total nitrogen storage changes had similar temporal patterns, with an initial decline during the early stage after afforestation, a gradual return of storage to the pre-afforestation levels, and then final net gains. Chen et al. (2017) reported that the conversion from evergreen broadleaved forests to Chinese fir plantations in the subtropical region of China caused STN to decrease in early stage, but to be finally restored after subtropical forest conversion. A change in STN is also a balance between nitrogen input and output. In the early stage of afforestation, nitrogen demand is high for rapid growth. If there is no sustainable nitrogen input, increasing the uptake of nitrogen for the rapid growth of trees will lead to a decrease in STN. As stand age increases, the decrease of growth rate leads to the decline of nitrogen uptake. If the nitrogen input can be maintained or increased, this will result in the accumulation of soil nitrogen.

**Figure 6:**
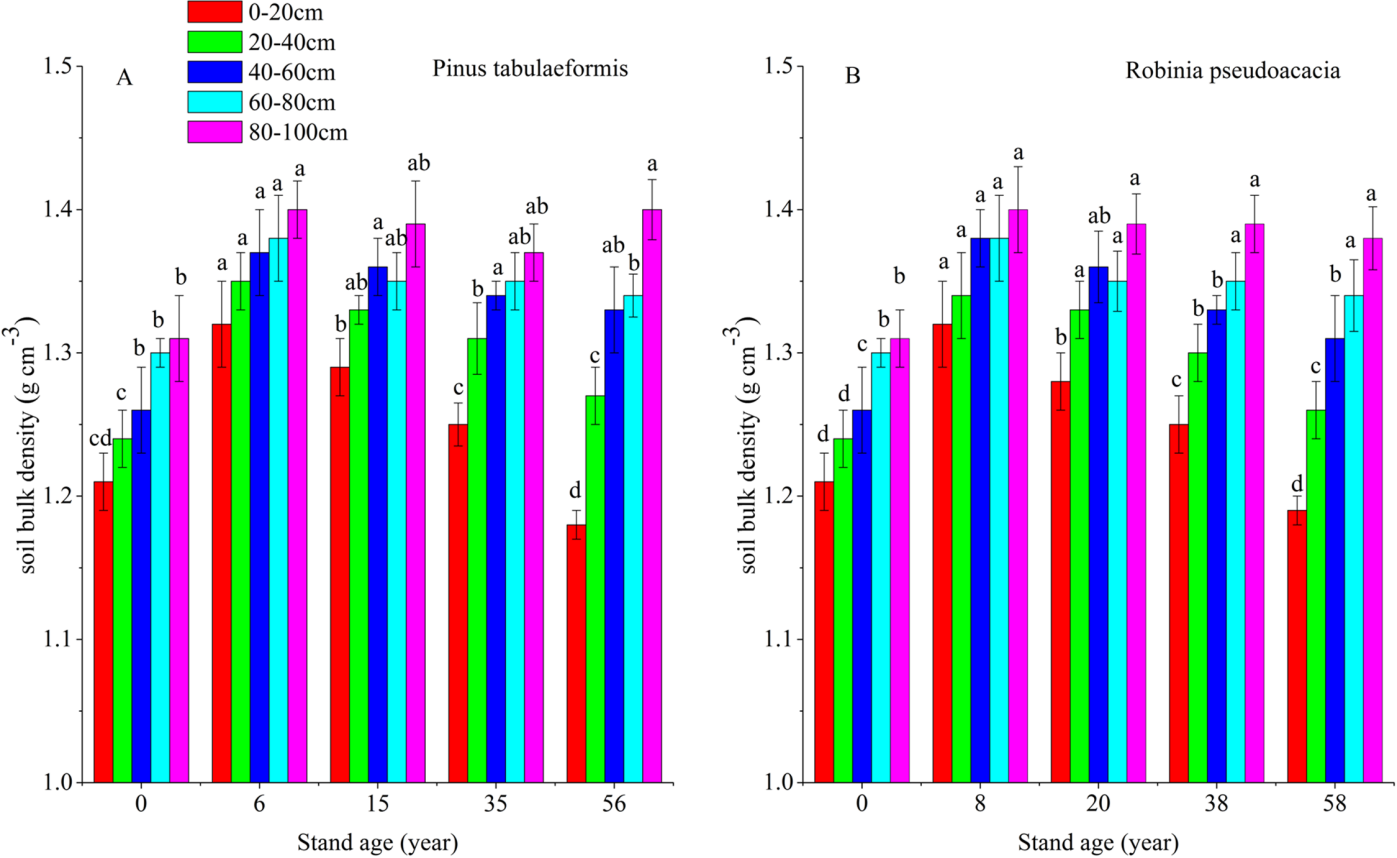
Soil bulk densities (BD) in different soil layers at each stand age of *Pinus tabulaeformis* and *Robinia pseudoacacia*. (A) Soil bulk density of *Pinus tabulaeformis*. (B) Soil bulk density of *Robinia pseudoacacia*. Note: Different letters indicate significant differences in the same soil layer of different stand ages at the 0.05 level.

Soil organic carbon storage is determined by SOC content, soil BD and soil depth. In this study, soil depth was fixed at 0–20, 20–40, 40–60, 60–80, and 80–100 cm. Therefore, SOC content and soil BD determine soil carbon storage. Afforestation breaks up soil aggregates, decreases soil porosity and accelerates composition and mineralization of soil organic matter (SOM) due to exposure of previously accessible SOM to microbial attack. In addition, litter was scarce in the early stage of afforestation, decreasing SOM input into the soil (Wang et al., 2011a; Deng, Shangguan & Sweeney, 2013). This resulted in a reduction of SOC and STN and an increase of soil BD during the early stage after converting farmland to forest. With the development of forest, SOC and soil porosity increased, resulting in a reduction of soil BD (Fig. 6). Some researchers have reported that SOC and STN have a negative relationship with soil BD during the restoration of degraded farmland (Wang et al., 2011b; Singh et al., 2012), and our results agreed with them (Figs. 2, 3 and 6). In our study, the change of soil BD partly indicated the trend of SOC and STN.

This study showed that SOC and STN content and storage decreased with increasing soil depth in the two types of plantations. The soil carbon and nitrogen were reserved mainly in the surface layer (0–20 cm). With tree growth, the SOC and STN drastically changed in the 0–20, 20–40, and 40–60 cm soil layers and were significantly different among these layers. These trends are consistent with other studies (Zhang et al., 2010; Li & Shao, 2014). The SOC storage in the 0–100 cm soil layer of the two plantations at different ages was similar to the estimates of 88.97 and 71.41 Mg ha^−1^ for *Quercus* and *R. pseudoacacia* forests of the approximate stand age on the Loess Plateau (Song et al., 2016). Moreover, our estimates were higher than the results of 26.3 Mg ha^−1^ for woodland in the semiarid catchment of the Loess Plateau, lying north of our site with a dryer climate (Li et al., 2013). This result is related to soil type, rainfall and human disturbance, among other factors. Li et al. (2014) estimated the STN storage of forest land in northeast China, which was 3.5 Mg ha^−1^ in the 0–20 cm depth and 12.9 Mg ha^−1^ in the 0–100 cm layer. The STN storage in our study was below the STN storage in northeast China estimated by Li et al. (2014). Considering that their study site had higher rainfall, our estimates may only represent the woodlands in semi-arid areas of the Loess Plateau.

In this study, the variation range of SOC content under various stand ages at 0–100 cm depth were 3.42–8.08 g kg^−1^ for the *P. tabulaeformis* forest and 3.94–9.41 g kg^−1^ for the *R. pseudoacacia* plantation, respectively. These results were higher than those of 2.2–6.3 g kg^−1^ for a poplar plantation lying north of our site with a dryer climate (Li et al., 2013). Our results are lower than the report for four other semiarid mature plantation forests, which are in the range of 7–55 g kg^−1^ (Gao et al., 2014). This may be due to the high stand density (2,830 ± 256 plants ha^−1^) of the *P. tabulaeformis* plantation, which could maximize tree and litter biomass. The SOC contents in this study were also lower than those found Song et al.’s (2016) study which was conducted in the northern Loess Plateau with loess soil in two near-mature forest types. This result suggests that SOC contents are subject to large variations and tend to increase with developing years.

In our study, the soil carbon sink capacity of the *R. pseudoacacia* forest was slightly higher than that of the *P. tabulaeformis* forest, but the difference was not significant between the two forests. Deng, Liu & Shangguan (2014) claimed that tree species played a significant role in determining the rate of change in soil carbon. Moreover, most reviews agree that converting cropland to conifer forest has a greater effect on soil carbon than a conversion to broadleaf forest (Guo & Gifford, 2002; Li, Niu & Luo, 2012). Differences in SOC sequestration between the two tree species may be attributable to the various C input or output patterns (Jandl et al., 2007). These differences may be related to their biomass allocation strategies and litter quality. Litter fall from the broadleaf plantation decayed faster than that of the conifer plantation due to differences in chemical composition and microclimatic condition (Gao et al., 2014).

### The relationship between SOC and STN

In our study, changes in SOC and STN were significantly correlated and had a similar pattern (Fig. 5). SOC and STN storage decreased initially during the early stage after afforestation, followed by a gradual return to pre-afforestation values, and then an increase to net gains of carbon and nitrogen (Figs. 2 and 3). Several researchers have indicated that nitrogen dynamics is a key parameter in the regulation of long-term terrestrial carbon sequestration (Rastetter, Agren & Shaver, 1997; Luo et al., 2004). Li, Niu & Luo (2012) indicated that soil carbon storage dynamics are closely coupled with nitrogen storage dynamics, as clearly demonstrated by the strong correlation between carbon and nitrogen changes. Hungate et al. (2003) found that the maintenance of soil carbon sequestration ability by afforestation mainly depends on the availability of nitrogen. Luo et al. (2004) suggested that the terrestrial ecosystem will become increasingly nitrogen-limited or will undergo progressive nitrogen limitation if terrestrial carbon is not accompanied by a simultaneous nitrogen gain. In this study, SOC was positively correlated with STN as stand age increased (Fig. 5), suggesting that soil nitrogen incrementally stimulated the accumulation of soil carbon. This is likely due to the increase in nitrogen after afforestation reducing nitrogen limitation and supporting long-term carbon sequestration (Hagedorn et al., 2012; Li, Niu & Luo, 2012). On the other hand, the enhancement of tree biomass production after afforestation will lead to a lack of STN and an increase of SOC in a few years because of the return of enhanced biomass production. In turn, the accumulation of SOC with forest development promotes ecosystem nitrogen retention, resulting in the accumulation of STN (Lewis, Castellano & Kaye, 2014).

The increased vegetation biomass and decreased soil erosion in long-term vegetation restoration lead to an increase in aboveground and underground carbon inputs, which may be the main factors contributing to soil carbon and nitrogen sequestration (Fu et al., 2000; Nelson, Schoenau & Malhi, 2008; Wang et al., 2016). We should pay more attention to changes in soil carbon and nitrogen reserves during long-term vegetation restoration.

## Conclusions

Forest ecosystem carbon and nitrogen vary with afforestation development. SOC is the main contributor to forest ecosystem carbon sequestration. The carbon sequestration rate of the *R. pseudoacacia* plantation ecosystem was slightly higher than that of the *P. tabulaeformis* plantation ecosystem. In both plantation forests, SOC and STN all showed an initial decrease during the early stage, followed by an increase to net carbon gains. Soil carbon and nitrogen changed with soil depth during tree development; the greatest SOC and STN were in the 0–20 cm topsoil, but they drastically decreased in the 0–60 cm profile. Below 60 cm, there was no significant difference at different stand ages. Future studies should take into account changes in soil carbon and nitrogen sequestration during long-term restoration in the Loess Plateau.

## Supplemental Information

10.7717/peerj.7708/supp-1 Supplemental Information 1 Soil organic carbon content and organic carbon storage in the different soil layers at each stand age of *Robinia pseudoacacia*.

10.7717/peerj.7708/supp-2 Supplemental Information 2 Soil nitrogen content and storage in the different soil layers at each stand age of *Robinia pseudoacacia*.

10.7717/peerj.7708/supp-3 Supplemental Information 3 The C/N ratios in different soil layers at each stand age of *Robinia pseudoacacia*.

10.7717/peerj.7708/supp-4 Supplemental Information 4 Soil organic carbon content and organic carbon storage in the different soil layers at each stand age of *Pinus tabulaeformis*.

10.7717/peerj.7708/supp-5 Supplemental Information 5 Soil nitrogen content and storage in the different soil layers at each stand age of *Pinus tabulaeformis*.

10.7717/peerj.7708/supp-6 Supplemental Information 6 The C/N ratios in different soil layers at each stand age of *Pinus tabulaeformis*.

## Additional Information and Declarations

### Data Availability

The following information was supplied regarding data availability: The raw data is available in the Supplemental Files.
